# Role of 5-Aminolevulinic Acids in Brain Tumor Surgeries in Pediatrics: A Systematic Review of Case Reports and Series

**DOI:** 10.7759/cureus.100532

**Published:** 2025-12-31

**Authors:** Alaa N Turkistani, Thamer H Alsharif, Badr E Hafiz, Ahmed Khawjah, Manar Alroidan, Abdulrahman D Alofi, Abdulaziz A Basurrah, Ahmed Z Awan, Mohammed Aref

**Affiliations:** 1 Section of Neurosurgery, Department of Neurosciences, King Faisal Specialist Hospital and Research Centre, Jeddah, SAU; 2 Department of Medicine, Royal College of Surgeons in Ireland, Dublin, IRL; 3 Department of Neurosurgery, King Abdulaziz University Hospital, Jeddah, SAU; 4 College of Medicine, Royal College of Surgeons in Ireland, Dublin, IRL; 5 Department of Neurosurgery, King Abdulaziz Specialist Hospital in Taif, Taif, SAU; 6 Department of Neurosurgery, King Abdullah Medical City, Makkah, SAU; 7 College of Medicine, King Abdulaziz University Faculty of Medicine, Jeddah, SAU

**Keywords:** 5-aminolevulinic acid, brain neoplasm, fluorescence, glioblastoma, medulloblastoma

## Abstract

Pediatric brain tumors remain a leading cause of cancer-related mortality in children, and achieving complete tumor resection is critical for improving survival and neurological outcomes. Fluorescence-guided surgery using 5-aminolevulinic acid (5-ALA) has emerged as a promising adjunct to enhance intraoperative visualization of tumor margins and improve surgical precision in pediatric patients. This systematic review evaluates the safety and efficacy of 5-ALA-guided surgery in pediatric brain tumors, with emphasis on fluorescence visibility, extent of resection, surgical morbidity, and oncological outcomes. Following Preferred Reporting Items for Systematic Reviews and Meta-Analyses (PRISMA) guidelines, a comprehensive literature search was conducted across PubMed, Embase, Medline, and Web of Science, identifying 19 studies reporting pediatric cases undergoing 5-ALA-assisted tumor resection. Data regarding patient demographics, tumor characteristics, surgical outcomes, recurrence rates, and complications were extracted and analyzed. A total of 282 pediatric patients with a mean age of 13 years were included, with glioblastomas, medulloblastomas, astrocytomas, and ependymomas being the most common tumor types. Intraoperative fluorescence was observed in 72% of cases, contributing to improved tumor delineation and facilitating more complete resections. Gross total resection was achieved in 77% of patients, of whom 85% demonstrated no tumor recurrence during follow-up periods ranging from three months to five years. Surgical complications occurred in approximately 10% of cases and were predominantly transient neurological deficits. Tumor recurrence was reported in 8% of patients, while overall mortality was 5%, largely associated with aggressive tumor subtypes. Overall, 5-ALA was well tolerated with minimal adverse effects. These findings suggest that 5-ALA fluorescence-guided surgery is a safe and effective adjunct in pediatric brain tumor surgery, enhancing tumor visualization and the extent of resection. Further prospective studies are warranted to establish pediatric-specific protocols and to optimize long-term surgical and oncological outcomes in this vulnerable population.

## Introduction and background

Pediatric brain tumors are among the most challenging and significant health concerns in children, representing the leading cause of cancer-related mortality in this age group [1]. These tumors included a wide range of histologies, with medulloblastomas, gliomas, and ependymomas being the most prevalent [2]. Advances in surgical techniques have improved survival outcomes, but achieving maximal tumor resection while minimizing neurological deficits remains a critical goal [3]. Fluorescence-guided surgery using 5-aminolevulinic acid (5-ALA) has emerged as a promising tool to enhance visualization of tumor margins, potentially improving surgical outcomes and reducing residual disease [4].

The use of 5-ALA in brain tumor surgeries is well-established in adult populations, mainly for high-grade gliomas, where it has been shown to significantly increase the extent of tumor resection and improve progression-free survival [5]. However, pediatric studies remain limited, with few case reports, series, and retrospective analyses evaluating its role in children. Some studies suggest that fluorescence positivity may vary significantly among different pediatric tumor types, necessitating further investigation to optimize its use in this population [6-9].

Despite the growing interest in 5-ALA-guided surgery, there are several gaps in the current literature. Many of the existing studies lack standardized protocols for 5-ALA administration in pediatric populations, leading to inconsistencies in outcomes [6,10,11]. There is also limited data on long-term follow-up, including recurrence rates and postoperative neurological deficits, which are critical for understanding the comprehensive benefits and risks of this approach.

This systematic review aims to address these gaps by consolidating and analyzing the available evidence on the role of 5-ALA in pediatric brain tumor surgeries. By focusing on published case reports, case series, and retrospective studies, we seek to provide a clearer understanding of the efficacy, safety, and challenges associated with 5-ALA in this unique patient population.

## Review

This systematic review was conducted following the guidelines outlined in the Preferred Reporting Items for Systematic Reviews and Meta-Analyses (PRISMA) statement. 

Study screening and selection

**Figure 1:**
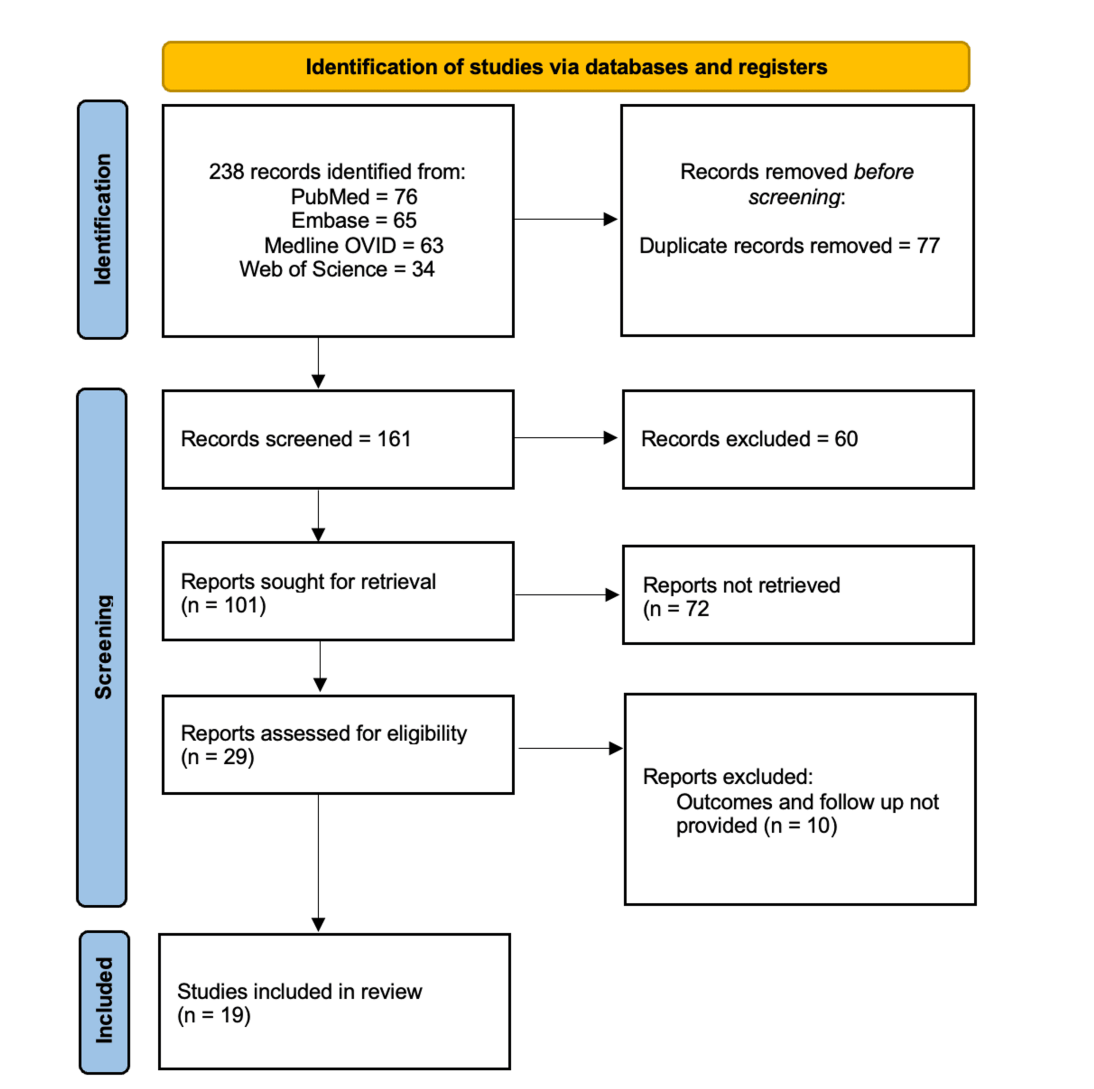
Preferred Reporting Items for Systematic Reviews and Meta-Analyses (PRISMA) flow chart showing study selection and inclusion

A comprehensive search was performed across PubMed, Embase, Medline OVID, and Web of Science databases using predefined keywords. The search strategy included three main domains: population (Pediatrics OR Children OR Adolescents), condition (Brain Tumor OR Brain Neoplasms OR Brain Cancer OR Intracranial Tumor), and intervention (5-Aminolevulinic Acid OR 5-ALA). The initial search yielded 238 records, and 77 duplicate entries were removed. Subsequently, 161 studies underwent screening based on titles and abstracts. From these, 101 full-text articles were retrieved for detailed evaluation. After excluding studies lacking relevant outcomes or follow-up data, 19 studies were deemed eligible for inclusion in the review (Figure 1).

Inclusion and exclusion criteria

Studies were included if they involved pediatric patients undergoing brain tumor surgeries with the application of 5-ALA, provided clinical outcomes related to tumor resection, and documented follow-up data. Case reports, case series, and retrospective studies were considered if they described surgical outcomes in detail. Exclusion criteria encompassed studies involving adult populations, those without sufficient outcome measures, and reports lacking clarity in follow-up or methodology.

Data extraction and synthesis

Data extraction was performed systematically, focusing on study characteristics (author, year, study design), patient demographics (age, gender, diagnosis), surgical details (type of procedure, use of 5-ALA), and outcomes (extent of resection, fluorescence positivity, and postoperative results). The extracted data were synthesized narratively to highlight trends in the use of 5-ALA and its impact on surgical outcomes in pediatric brain tumors.

Outcome measures

The primary outcome measure was the extent of tumor resection achieved using fluorescence-guided surgery with 5-ALA. Secondary outcomes included fluorescence positivity rates, postoperative complications, recurrence rates, and long-term follow-up findings such as neurological outcomes and survival. Quantitative data were tabulated, while qualitative findings were synthesized to provide a comprehensive overview of the role of 5-ALA in this setting. 

Results

This systematic review and meta-analysis included 19 studies [12-30], comprising case reports, case series, and retrospective analysis, which provided data about the demographics, diagnosis, and surgical outcomes in pediatric patients undergoing brain tumor surgeries with 5-ALA. Patient ages ranged from six months to 19 years, with a median age of 13 years. Among the 282 patients included, there was a slight male predominance, with 161 male patients (57%) and 121 female patients (43%). Diagnoses were diverse, with glioblastomas, medulloblastomas, astrocytomas, and ependymomas being the most frequently reported (Table 1).

**Table 1:** Study characteristics and participants’ demographics N/A: not available, PNET: primitive neuroectodermal tumor, SD: standard deviation

Author (year)	Type/design	No. of cases	Diagnosis	Age (years)	Gender
Barbagallo et al. (2014) [12]	Case series	3	Glioblastoma (2), medulloblastoma (1)	12.67 (SD = 5.03)	Female (1), male (2)
Agawa and Wataya (2018) [13]	Case report	1	Astroblastoma	13	Female
Eicker et al. (2011) [14]	Case report	1	Medulloblastoma	15	Female
Takeda et al. (2017) [15]	Case report	1	Mixed germ cell tumor	16	Male
Beez et al. (2014) [16]	Case series	16	Astrocytoma (8), glioblastoma (3), ependymoma (1), medulloblastoma (4)	8.94 (SD = 4.79)	Female (10), male (6)
Ruge et al. (2009) [17]	Case report	1	Pleomorphic xanthoastrocytoma	9	Female
Takeda et al. (2022) [18]	Retrospective study	4	Glioblastoma (1), medulloblastoma (1), astrocytoma (1), pineoblastoma (1)	7.12 (SD = 5.14)	Female (1), male (3)
Bernal et al. (2015) [19]	Case report	1	Meningeal sarcoma	7	Male
Schwake et al. (2019) [20]	Case series	11	Astrocytoma (5), pleomorphic xanthoastrocytoma (1), medulloblastoma (3), ependymoma (2),	8.8 (SD = 5.21)	Female (6), male (5)
Labuschagne (2020) [21]	Case series	19	Medulloblastoma (9), ependymoma (10)	5.16 (SD = 3.2)	Female (8), male (11)
Preuß et al. (2013) [22]	Retrospective study	18	Medulloblastoma (4), glioblastoma (3), supratentorial PNET (1), neuroblastoma (1), ependymoma (2), astrocytoma (4), ganglioglioma (3)	10.94 (SD = 4.67)	Female (5), male (13)
Fudaba et al. (2020) [23]	Case report	1	Astroblastoma	6	Female
Mui et al. (2023) [24]	Case report and literature review	1	Ependymoma	4	Female (2), male (6)
Labuschagne (2020) [25]	Retrospective study	8	Astrocytoma (8)	6.12 (SD = 4.22)	Female (2), male (6)
Milos et al. (2022) [26]	Case series	14	Medulloblastoma (4), astrocytoma (6), glioblastoma (1), meningioma (1), DIPG (1), oligodendroglioma (1)	7.78 (SD = 4.13)	Female (6), male (8)
Skjøth-Rasmussen et al. (2015) [27]	Case report	1	Medulloblastoma	9	Male
Maeda et al. (2023) [28]	Case report	1	Pleomorphic xanthoastrocytoma	14	Female
Roth and Constantini (2017) [29]	Case series	14	Astrocytoma (6), medulloblastoma (2), dysembryoplastic neuroepithelial tumor (1), hemangiopericytoma (2), ganglioneuroblastoma (1), oligodendroglioma (1), ganglioglioma (1)	11.86 (SD = 2.77)	Female (1), N/A (13)
Stummer et al. (2014) [30]	Retrospective study	78	Glioblastomas, astrocytomas, ependymomas, PNETs, gangliogliomas and medulloblastomas	Median 13 (range: 1.6-17)	Female (27), male (51)

Common clinical presentations in the majority of cases were headache, nausea, and vomiting. A few cases also reported variable symptoms such as dizziness, polyuria, seizures, hand bruising, etc. Surgical approaches included gross total resection (GTR) in the majority of cases, aiming for maximal tumor removal with minimal neurological deficits. Among the patients, 216 (77%) achieved GTR, while 66 underwent subtotal or near-total resection. Tumors with different grades ranging from grade I to grade IV, showing variable pathologies, less common pathologies include medulloblastoma, germinoma, sarcoma, and gliomas (Table 2).

**Table 2:** Histology and surgical approaches for different clinical presentations DIPG: diffuse intrinsic pontine glioma, INSS: International Neuroblastoma Staging System, N/A: not available

Author (year)	Clinical presentation	Surgical approach	Pathology
Barbagallo et al. (2014) [12]	Intracranial hypertension, cognitive deficits, dysphasia, acalculia, dysgraphia, dyslexia, headache, vomiting, drowsiness, and gait disturbance	Gross total resection (1), right-sided temporal craniotomy (1), suboccipital median craniectomy (1)	Grade IV (2), desmoplastic medulloblastoma (1)
Agawa and Wataya (2018) [13]	Headache, vomiting, nausea, and visual hallucinations	Gross total resection	Low-grade
Eicker et al. (2011) [14]	Headache, vomiting, nausea, and dizziness	Gross total resection	Grade IV
Takeda et al. (2017) [15]	Headache and polyuria	Radiation therapy, chemotherapy, followed by total resection of tumor	Germinoma with a typical two-cell pattern and was positive for placental alkaline phosphatase
Beez et al. (2014) [16]	Headache, vomiting, gait disturbance, generalized seizures and coma	Near total resection (3), gross total resection (6), subtotal resection (7)	Grade I (7), Grade III (2), Grade IV (7)
Ruge et al. (2009) [17]	Complex partial seizures	A right temporal craniotomy	Grade II
Takeda et al. (2022) [18]	N/A	Partial resection (2), gross total resection (2)	N/A
García et al. (2015) [19]	Headache and diplopia. Bilateral sixth nerve palsy and bilateral papilledema	Left frontal parasagittal craniotomy	Meningeal sarcoma
Schwake et al. (2019) [20]	N/A	Gross total resection (8), subtotal resection (3)	Grade I (2), Grade II (2), Grade III (4), Grade IV (3)
Labuschagne (2020) [21]	Headache, vomiting, gait disturbance, ataxia and abducent nerve palsy	Gross total resection (15), near total resection (4)	Grade II (5), Grade IV (9), Grade III (5)
Preuß et al. (2013) [22]	N/A	Gross total resection (14), subtotal resection (2), small remnant (1), partial resection (1)	Grade I (7), Grade III (2), Grade IV (8), INSS 4 (1)
Fudaba et al. (2020) [23]	Painful head bruise	Left parietal osteoplastic craniotomy	Astroblastoma with focal anaplastic features and MN1 alteration
Mui et al. (2023) [24]	headache, lethargy and vomiting	Suboccipital craniotomy, C1 laminectomy	Grade III
Labuschagne (2020) [25]	N/A	Subtotal resection (2), near total resection (3), gross total resection (3)	Grade I (3), Grade II (3), Grade III (2)
Milos et al. (2022) [26]	Headache, vomiting and nausea	Partial resection (4), gross total resection (10)	Grade I (4), Grade II (4), Grade IV (5), DIPG (1)
Skjøth-Rasmussen et al. (2015) [27]	Severe headache, vomiting, gait disturbance, and left-sided weakness	Tumor resection	Grade IV
Maeda et al. (2023) [28]	Seizure, nausea, and phantosmia	Tumor resection	Pleomorphic xanthoastrocytoma
Roth and Constantini (2017) [29]	N/A	Tumor resection (14)	N/A (12), Grade 2 (2)
Stummer et al. (2014) [30]	Headache, nausea/vomiting, visual symptoms, paresis, and ataxia	Fluorescence-guided resections	Malignant gliomas, ependymomas, medulloblastomas, and astrocytomas

Fluorescence positivity using 5-ALA was reported across different tumor types, reflecting its utility in identifying tumor margins. Doses of 5-ALA ranged from 10 mg/kg to 31 mg/kg, administered orally or through a gastric tube two to six hours before surgery. Fluorescence intensity also varied, with heterogeneous patterns noted in a subset of cases. Tumors with high-grade histology, such as glioblastomas and medulloblastomas, had the highest fluorescence positivity rates, while low-grade tumors like astrocytomas showed variability. Of the 282 patients, 203 (72%) demonstrated positive fluorescence during surgery, facilitating better visualization of tumor tissue (Table 3).

**Table 3:** Fluorescence positivity with tumor grading N/A: not available

Author (year)	5-ALA	Dose	Intensity
Barbagallo et al. (2014) [12]	Orally 3 hours before surgery (3)	750 mg diluted in 200 mL of water (1), 560 mg diluted in 200 mL of water (1), 1000 mg diluted in 200 mL of water (1)	Positive (2), negative (1)
Agawa and Wataya (2018) [13]	Orally 3 hours before surgery	20 mg/kg dissolved in 50 mL of water	Positive
Eicker et al. (2011) [14]	Orally 3 hours before surgery	20 mg/kg	Positive
Takeda et al. (2017) [15]	Orally 3 hours before surgery	20 mg/kg	Positive
Beez et al. (2014) [16]	Orally 3-5 hours before surgery (16)	20 mg/kg dissolved in water (16)	Positive (3), negative (13)
Ruge et al. (2009) [17]	Orally 3-5 hours before surgery	10 mg/kg dissolved in water	Positive
Takeda et al. (2022) [18]	Orally 3 hours before surgery (4)	20 mg/kg (4)	Positive (2), negative (2)
Bernal García et al. (2015) [19]	Orally 3 hours before surgery	20 mg/kg	Positive
Schwake et al. (2019) [20]	Orally 4 hours prior to surgery (11)	N/A (11)	Positive (6), negative (5)
Labuschagne (2020) [21]	Orally 4 hours prior to surgery (19)	20 mg/kg suspended in 50 mL of tap water (19)	Positive (18), negative (1)
Preuß et al. (2013) [22]	Orally 2 hours prior to surgery (18)	20 mg/kg (18)	Positive (10), negative (8)
Fudaba et al. (2020) [23]	N/A	N/A	N/a
Mui et al. (2023) [24]	Administered on the morning of surgery	20 mg/kg, pt is 19 kg	Positive
Labuschagne (2020) [25]	Orally 4 hours prior to surgery (8)	20 mg/kg suspended in 50 mL of tap water (8)	Positive (5), negative (3)
Milos et al. (2022) [26]	Gastric tube 3 hours prior to surgery (7), Orally 3-4 hours prior to surgery (7)	20 mg/kg pt is 16 kg dissolved in 9 mL (7), 20 mg/kg pt is 25 kg dissolved in 50-100 mL (7)	Positive (13), negative (1)
Skjøth-Rasmussen et al. (2015) [27]	Orally 3 hours before surgery	20 mg/kg	Positive
Maeda et al. (2023) [28]	Orally 3-4 hours prior to surgery	20 mg/kg	Positive
Roth and Constantini (2017) [29]	Orally 3-6 hours before surgery (14)	20-32 mg/kg, suspended in 50 cm³ of tap water (14)	Positive (1), positive/ heterogeneous (2), negative (4), N/A (7)
Stummer et al. (2014) [30]	Daily 2-6 hours before surgery	20 mg/kg body weight	Positive/heterogeneous

Postoperative outcomes were assessed in terms of tumor resection and subsequent complications. Postoperative complications were infrequent, with transient neurological deficits such as cerebellar mutism, ataxia, or cranial nerve palsies being reported in approximately 10% of cases. The effectiveness of fluorescence-guided surgery with 5-ALA was further demonstrated by follow-up data. Among patients with GTR, 85% had no evidence of recurrence at follow-up intervals ranging from three months to five years. Long-term follow-up data revealed recurrence rates of 8% and mortality rates of 5%, predominantly in patients with high-grade tumors. Postoperative imaging confirmed successful tumor removal in most cases, with hydrocephalus and residual disease being rare complications. Some studies also reported no adverse events directly attributable to 5-ALA, highlighting its safety profile in pediatric populations (Table 4).

**Table 4:** Postoperative outcomes following fluorescence-guided surgery N/A: not available

Author (year)	Outcome	Follow-up	Follow-up duration	Postoperative symptoms
Barbagallo et al. (2014) [12]	Gross total resection (2), partial resection (1)	Cognitive impairment improved (1), no improvement (1), gradual improvement (1)	7 months (2), 1 year (1)	N/A
Agawa and Wataya (2018) [13]	Gross total resection	No recurrence	1 year	N/A
Eicker et al. (2011) [14]	Gross total resection	Uneventful	N/A	N/A
Takeda et al. (2017) [15]	Gross total resection	No recurrence	N/A	N/A
Beez et al. (2014) [16]	Near total resection (3), gross total resection (6), subtotal resection (7)	N/A (16)	N/A (16)	Oculomotor and facial nerve palsy, cerebellar mutism, hemiparesis, abducent nerve palsy
Ruge et al. (2009) [17]	Gross total resection	Uneventful	6 months	Uneventful
Takeda et al. (2022) [18]	Partial resection (1), gross total resection (3)	Uneventful (3), leakage of cerebrospinal fluid postop (1)	N/A (4)	Uneventful
Bernal García et al. (2015) [19]	Gross total resection	No recurrence	5 years	Uneventful
Schwake et al. (2019) [20]	Gross total resection (8), subtotal resection (3)	Uneventful (11)	N/A (11)	Uneventful (11)
Labuschagne (2020) [21]	Gross total resection (15), near total resection (4)	Posterior fossa syndrome (1), transient CN VI palsy (1), transient worsening of ataxia (1), uneventful (16)	N/A (19)	N/A
Preuß et al. (2013) [22]	Gross total resection (14), subtotal resection (2), near total resection (1), partial resection (1)	No recurrence (12), meningeal/multiple lymph nodes at 13 months (1), lost to follow-up (2), disseminated and contralateral at 8 months (1), dead at 25 months. Contralateral at 21 months (1), local and intracranial recurrence (1)	2 months-35 months	Posterior fossa syndrome, gait ataxia, temporal left epilepsy
Fudaba et al. (2020) [23]	Gross total resection	No recurrence	27 months	Uneventful
Mui et al. (2023) [24]	Gross total resection	Noted to be neurologically intact aside from a very subtle weakness of his left hand.	3 months	Spontaneous intraparenchymal hemorrhage 48 hours postoperatively
Labuschagne (2020) [25]	Subtotal resection (2), near total resection (3), gross total resection (3)	No permanent neurological deficits (8)	3 months	Uneventful (8)
Milos et al. (2022) [26]	Partial resection (4), Gross total resection (10)	N/A (14)	N/A (14)	Neurological deficits postoperatively (cerebellar mutism, left leg paresis, right hemiparesis) (3)
Jane Skjøth-Rasmussen et al. (2015) [27]	Gross total resection	No tumor relapse	24 months	No improvement
Maeda et al. (2023) [28]	Gross total resection	Seizures and phantosmia became less frequent	N/A	N/A
Roth and Constantini (2017) [29]	N/A (12), Gross total resection (2)	N/A (13), death (1)	N/A (13), 1 month (1)	N/A (13), diffuse rash, fever, and leukocytosis (1)
Stummer et al. (2014) [30]	Gross total resection	N/A	N/A	Transient hoarseness, cerebellar neurological deficits, and infected shunts

This systematic review has explored data from 19 studies regarding the role of 5-ALA in pediatric brain tumor surgeries, which has shown the efficacy, safety, and challenges of using fluorescence-guided techniques in this unique patient population. The ability of 5-ALA to enhance tumor visualization during surgery has been a significant advantage, as seen in the GTR achieved in 77% of patients included in the review. This represents an impressive improvement given the complex anatomy and histologies of pediatric brain tumors. Fluorescence positivity was observed in 72% of cases, underscoring the effectiveness of this technique in aiding surgeons to delineate tumor margins. These findings mirror similar successes observed in adult populations, especially for high-grade gliomas [31,32], but highlight the unique challenges in applying these results to the pediatric demographic.

Fluorescence positivity demonstrated marked variability across tumor types, with high-grade gliomas and medulloblastomas showing the highest rates of success. This is consistent with prior findings that link higher fluorescence intensity to tumor grade, as higher-grade tumors tend to accumulate more protoporphyrin IX [33,34]. However, the variability observed in low-grade tumors, such as astrocytomas, complicates the surgical approach, potentially leading to incomplete resections. Advances in imaging modalities, such as fluorescence spectroscopy, might address these inconsistencies and improve surgical precision. Such innovations would enable more reliable identification of tumor tissues, especially for lower-grade pediatric tumors that currently pose challenges in intraoperative visualization [35].

The safety of 5-ALA in pediatric populations was supported by the reviewed studies, which reported a low incidence of adverse effects directly attributable to the compound. Transient neurological deficits, such as cerebellar mutism, ataxia, and cranial nerve palsies, were noted in approximately 10% of cases. However, these complications were predominantly linked to surgical manipulation rather than the use of 5-ALA itself. Importantly, no significant long-term adverse effects were reported, reaffirming the safety profile of 5-ALA that has been established in adult neurosurgical practices. This favorable safety profile makes 5-ALA a viable option for fluorescence-guided surgery in pediatric patients, provided that its use is carefully monitored and tailored to individual patient needs.

Postoperative outcomes also underscored the benefits of 5-ALA-guided surgery. Among patients who achieved GTR, 85% exhibited no evidence of recurrence during follow-up periods ranging from three months to five years. This is a promising finding, particularly for high-grade tumors, where recurrence rates are typically higher. Additionally, the review highlighted a recurrence rate of 8% and a mortality rate of 5%, which compares favorably to outcomes in standard pediatric neurosurgery without fluorescence guidance. These results suggest that 5-ALA could play a pivotal role in improving long-term survival and reducing the burden of residual disease in pediatric brain tumor patients [36,37].

Despite these encouraging outcomes, the adoption of 5-ALA in pediatric neurosurgery is not without its challenges. The heterogeneity in dosing protocols, ranging from 10 mg/kg to 31 mg/kg, reflects a lack of standardization, which may affect both fluorescence consistency and surgical outcomes. Additionally, the close proximity of pediatric tumors to critical brain structures heightens the risk of incomplete resections and neurological complications. This underscores the need for precise intraoperative techniques and standardized protocols to optimize the use of 5-ALA in this population [30].

The differences in tumor biology between pediatric and adult populations further complicate the application of 5-ALA in children. For example, while adult studies frequently report high fluorescence positivity in gliomas, the fluorescence in pediatric low-grade tumors is often inconsistent. This variability is likely due to differences in tumor biology and protoporphyrin IX metabolism between children and adults. Consequently, pediatric-specific research is essential to fully understand these differences and optimize the application of 5-ALA in this demographic. Moreover, while adult studies have shown significant long-term survival benefits associated with 5-ALA-guided surgery [38], such outcomes are less well-documented in pediatric cohorts due to shorter follow-up durations.

The reviewed studies also revealed considerable heterogeneity in methodologies, including variations in the routes and timing of 5-ALA administration. At the same time, oral administration three to four hours before surgery was the most common approach; some cases employed gastric tube administration. These variations highlight the need for standardized protocols to enhance reproducibility and ensure consistent outcomes. Standardization would also facilitate more robust comparisons between studies and enable the development of evidence-based guidelines for 5-ALA use in pediatric neurosurgery [6,38].

Emerging techniques offer opportunities to further enhance the utility of 5-ALA in pediatric populations. For instance, advanced fluorescence spectroscopy and intraoperative imaging could improve the accuracy of tumor visualization, particularly in cases with heterogeneous fluorescence patterns [39]. Integrating 5-ALA with other imaging modalities, such as intraoperative MRI, might also refine surgical strategies by providing complementary data on tumor location and extent [40]. Prospective multicenter studies are needed to validate these innovations and establish best practices for their implementation in clinical settings.

The limitations of this review, including the retrospective nature of most included studies and the reliance on case reports and series, must be acknowledged. These factors introduce biases and limit the generalizability of the findings. The absence of randomized controlled trials in pediatric populations shows a significant barrier to drawing definitive conclusions about the efficacy of 5-ALA in this context. Addressing these gaps through well-designed prospective studies will be critical to advancing the field and ensuring that pediatric patients benefit fully from this promising technology.

## Conclusions

In pediatric brain tumor surgeries, the integration of 5-ALA can help increase the degree of tumor resection and provide better oncological results. Despite the fluorescence variability and methodological inconstancy, the technique remains safe and shows long-term optimistic outcomes. Standardization of protocols and further research into tumor-specific fluorescence patterns are essential to optimize its application. Integrating emerging imaging technologies with 5-ALA-guided surgery may pave the way for improved surgical precision and patient outcomes. Overall, 5-ALA is a helpful contribution to the neurosurgical practice in the pediatric brain tumor population.
